# Retention strategies are routinely communicated to potential trial participants but often differ from what was planned in the trial protocol: an analysis of adult participant information leaflets and their corresponding protocols

**DOI:** 10.1186/s13063-024-08194-7

**Published:** 2024-06-10

**Authors:** Ellen Murphy, Katie Gillies, Frances Shiely

**Affiliations:** 1 Health Research Board-Trials Methodology Research Network (HRB-TMRN), Galway, Ireland; 2 Trials Research and Methodologies Unit (TRAMS), Health Research Board Clinical Research Facility University College Cork, Cork, Ireland; 3 Health Services Research Unit, University of Aberdeen, Aberdeen, UK; 4 School of Public Health, University College Cork, Cork, Ireland

**Keywords:** Retention, Retention strategy, Reporting, Communication, Participant information leaflet, Patient information leaflet, Informed consent

## Abstract

**Background:**

Retaining participants in randomised controlled trials (RCTs) is challenging and trial teams are often required to use strategies to ensure retention or improve it. Other than monetary incentives, there is no requirement to disclose the use of retention strategies to the participant. Additionally, not all retention strategies are developed at the planning stage, i.e. post-funding during protocol development, but some protocols include strategies for participant retention as retention is considered and planned for early in the trial planning stage. It is yet unknown if these plans are communicated in the corresponding participant information leaflets (PILs). The purpose of our study was to determine if PILs communicate plans to promote participant retention and, if so, are these outlined in the corresponding trial protocol.

**Methods:**

Ninety-two adult PILs and their 90 corresponding protocols from Clinical Trial Units (CTUs) in the UK were analysed. Directed (deductive) content analysis was used to analyse the participant retention text from the PILs. Data were presented using a narrative summary and frequencies where appropriate.

**Results:**

Plans to promote participant retention were communicated in 81.5% (*n* = 75/92) of PILs. Fifty-seven percent (*n* = 43/75) of PILs communicated plans to use “combined strategies” to promote participant retention. The most common individual retention strategy was telling the participants that data collection for the trial would be scheduled during routine care visits (16%; *n* = 12/75 PILs). The importance of retention and the impact that missing or deleted data (deleting data collected prior to withdrawal) has on the ability to answer the research question were explained in 6.5% (*n* = 6/92) and 5.4% (*n* = 5/92) of PILs respectively. Out of the 59 PILs and 58 matching protocols that both communicated plans to use strategies to promote participant retention, 18.6% (*n* = 11/59) communicated the same information, the remaining 81.4% (*n* = 48/59) of PILs either only partially communicated (45.8%; *n* = 27/59) the same information or did not communicate the same information (35.6%; *n* = 21/59) as the protocol with regard to the retention strategy(ies).

**Conclusion:**

Retention strategies are frequently communicated to potential trial participants in PILs; however, the information provided often differs from the content in the corresponding protocol. Participant retention considerations are best done at the planning stage of the trial and we encourage trial teams to be consistent in the communication of these strategies in both the protocol and PIL.

**Supplementary Information:**

The online version contains supplementary material available at 10.1186/s13063-024-08194-7.

## Background

Retention in trials has been identified as a research priority in the UK [1]. It is estimated that up to 50% of trials have loss-to-follow-up rates, i.e. the proportion of participants failing to provide valid primary outcome data, exceeding 11% [2, 3]. The overall result of the trial can be different if the outcomes for those not retained are assumed in the opposite direction [4–6]. In high-impact journals, it was found that 25% of randomised controlled trials (RCTs) had a fragility index of 3 or less, meaning that the statistical significance of the results are lost if the outcomes for three participants are changed from not experiencing the primary outcome to experiencing the primary outcome [6, 7]. Even low rates of loss-to-follow-up can cause issues for trial validity as the generalisability, the reliability and the confidence in the trial findings are affected [5, 6, 8, 9]. This contributes to research waste [10–12]. To avoid these issues, the optimum solution is to retain the trial participants [4].

Retaining participants in RCTs however is challenging [3, 4] and trial teams are often required to use strategies to ensure retention or improve it [3, 13]. Some of these strategies are implemented at the trial recruitment stage, e.g. video-enhanced patient information versus standard information or providing a pen at recruitment versus no pen; however, there is little evidence to support the use of these strategies or retention strategies in general [3]. Research shows that at the recruitment stage, retention information is poorly communicated, if it is communicated at all, both in participant information leaflets [14] and in trial recruitment consultations [15]. There is also an imbalance between retention information and information on the right to withdraw, with a greater focus on participant withdrawal [14, 15]. Providing a greater balance between the positives and negatives of trial participation may help aid participant retention as participants are more informed about trial expectations [16]. It has been highlighted that some participants do not complete follow-up data collection due to the belief that their individual contribution did not make a difference to the trial [17]. Additionally, in trial recruitment consultations, it was found that potential trial participants were not provided with the opportunity to talk about aspects of trial retention that may be important regarding their initial decision to participate in the trial [15]. Fixing this information imbalance may help to improve retention in trials [14].

Outlining information during informed consent helps to set participants’ trial expectations [18–20] as poor expectation setting may hinder retention [20]. Participant information leaflets (PILs) are required by regulation to outline information about the trial such as the purpose of the trial, treatment information, the procedures involved, the anticipated risks and benefits and information about data safety and handling [21, 22]. There is also an ethical and moral obligation to do so [23]. This information should include activities related to promoting retention if such activities are planned by trial teams. Disclosing information on monetary compensation and other supports, e.g. travel, meals, child-care and compensation for loss of earnings, is recommended [21, 24]. Apart from these forms of compensation, there are a plethora of strategies that can be used to promote participant retention [3]. However, there is no recommendation or regulation on the need to disclose them to the participant and evidence on whether trial teams should, or not, is lacking. Additionally, some but not all strategies to promote participant retention are developed at the planning stage, i.e. post-funding during protocol development [25, 26]; therefore, we would expect these plans to be outlined in PILs.

It is also recommended to include plans for promoting participant retention in protocols as per the Standard Protocol Items: Recommendations for Interventional Trials (SPIRIT) 2013 guidelines item 18b “plans to promote participant retention and complete follow-up, including list of any outcome data to be collected for participants who discontinue or deviate from intervention protocols” ([27]:3). Previous research shows that 36.8% of RCT protocols include proactive plans to use strategies to promote participant retention [26], but it is yet unknown if the retention strategy(ies) outlined in the protocol are communicated in the corresponding PILs.

The purpose of this study is twofold: to establish if trial teams communicate retention strategies in PILs, and if they do, to establish if the retention strategy(ies) were outlined in the corresponding trial protocol.

## Methods

### Participant information leaflet and protocol selection

Our starting point was the PILs. As part of a previous research project, we collected PILs (*n* = 214) from Clinical Trial Units in the UK and Ireland (see the “Acknowledgements” section). Given our intention was to compare the PIL to the trial protocol, the time period of interest was from 2014 to the present, because guidelines on protocol development for trials, SPIRIT guidelines, were published in 2013, and they contain an item on retention (item 18b) [27, 28]. We permitted a one-year grace period to allow for uptake by trialists. There were 185 PILs corresponding to 144 protocols available in this time period. We sought the corresponding trial protocols and located 112 which were publicly available. We emailed Trial Managers and Chief Investigators to seek the remaining 32 protocols. Where a response was not received and/or the protocol could not be obtained, we excluded the PIL from the analysis. Thus, 157 PILs and their 121 corresponding protocols were available for analysis. These numbers are different because some PILs corresponded to the same trial protocol, for example if a trial had a separate child and parent PIL. Our PIL repository contained PILs for both adult and paediatric RCTs. We divided the PILs into two groups: group 1—adult PILs, for adults with the capacity to make a decision for themselves, i.e. those who can provide their own consent (*n*=92); group 2—child PILs, the corresponding parent PILs, legal/personal representative PILs and PILs for adults in research without prior consent. This study reports on the analysis of the 92 adult PILs and their 90 corresponding protocols.

### Data extraction

The data to be extracted from both the PILs and the protocols was informed by a scoping review on the communication of retention strategies in trial protocols [26], and a prior study on reducing attrition in trials [14]. These were agreed by all authors and are shown in Table 1. In this project, we are specifically interested in SPIRIT item 18b “plans to promote participant retention and complete follow-up, including list of any outcome data to be collected for participants who discontinue or deviate from intervention protocols” ([27]:3). This was broken down into three parts: SPIRIT item 18b(i), plans to promote participant retention; item 18b(ii), plans to complete follow-up including list of any outcome data to be collected for participants who *discontinue* from intervention protocols; and item 18b(iii), plans to complete follow-up including list of any outcome data to be collected for participants who *deviate* from intervention protocols [27, 28].

**Table 1:** Data extracted from the participant information leaflets

**Study characteristics**	Trial name
	Year of the PIL
	Study design—cluster or individually randomised
	Phase of the trial
	Funding status
	Clinical speciality
	Planned sample size
	Study population
	Intervention type
	Mode of follow-up
	Primary outcome
**SPIRIT item 18b information**	**Item 18b—**“plans to promote participant retention and complete follow-up, including list of any outcome data to be collected for participants who discontinue or deviate from intervention protocols” ([27]:3)
	**Does the PIL report item 18b(i)—**plans to promote participant retention (yes or no)
	**Does the PIL report item 18b(ii)—**plans to complete follow-up including list of any outcome data to be collected for participants who *discontinue* from intervention protocols (yes or no)
	**Does the PIL report item 18b(iii)—**plans to complete follow-up including list of any outcome data to be collected for participants who *deviate* from intervention protocols (yes or no)
**Retention information**	Does the PIL explain the importance of retention in the trial/the importance of continued follow-up in the trial (yes or no)
	Does the PIL explain the impact of missing or deleted trial data^a^ on the ability to answer the clinical/research question (yes or no)
**Mention of patient and public involvement (PPI) involvement in the trial in the PIL**	Does the PIL mention PPI involvement in the trial? (yes or no)
**Corresponding protocol content regarding SPIRIT item 18b**	
**Protocol characteristics**	Publication location (peer-reviewed journal or trial website)
**SPIRIT information**	Does the protocol report use the SPIRIT 2013 guidelines when developing the protocol? (yes or no)
**SPIRIT item 18b information**	**Does the protocol report item 18b(i) –** plans to promote participant retention (yes or no)
	**Does the protocol report item 18b(ii) –** plans to complete follow-up including list of any outcome data to be collected for participants who *discontinue* from intervention protocols (yes or no)
	**Does the protocol report item 18b(iii) -** plans to complete follow-up including list of any outcome data to be collected for participants who *deviate* from intervention protocols (yes or no)
**Mention of PPI in the trial in the protocol**	Does the protocol mention PPI in the trial? (yes or no)

^a^Deleting participant data collected prior to withdrawal [14]

The data extraction form was piloted by EM using a sample of 5 PILs and was reviewed by FS to ensure the data extracted best met the objectives of the project. EM carried out the data extraction. Double extraction of 10% (*n* = 9/92) of the total number of PILs was carried out by FS to ensure consistency and improve the reliability of the data extraction process. There were no disagreements. All extracted information was entered into a Microsoft Excel file.

### Data analysis

We analysed 92 PILs. PIL characteristics such as year, clinical speciality, mode of follow-up, sample size, population and intervention type were summarised using frequencies. Directed (deductive) content analysis was used to analyse the text from the PILs as per the process outlined by Elo and Kyngas [29–32]. This process has three main phases; the preparation phase, the organisation phase and the reporting phase [31]. Data were presented using a narrative summary and frequencies where appropriate.

#### The preparation phase

Prior to starting the extraction process, for the purposes of this study, we defined a retention strategy as an activity/action that is conducted with the purpose of reducing missing data or improving data completeness. We did not extract information on improving adherence or compliance with an intervention. We also read the relevant literature on retention strategies in clinical trials [3, 13, 33]. This helped identify the types of retention strategies to look for during data extraction. The unit of analysis was the PIL. The meaning unit was defined as the textual unit within the PIL, i.e. the passage of text that represents a retention strategy.

### The organisation phase

From each PIL, we extracted any text that represented a retention strategy. All the meaning units were re-read and each of the passages was coded using predetermined codes which were derived from the Cochrane Review on strategies to improve retention in randomised trials [3], and retention strategies that were identified and routinely used by UK Clinical Trial Units [13]. These codes were then mapped to the ORRCA (Online Resource for Research in Clinical triAls) retention domains [33]. The ORRCA project brings together work in the field of recruitment and retention into searchable databases; the ORRCA retention domains are the key retention themes seen throughout the database [34]. Where multiple codes were identified within a single PIL, a new code was developed: “combined strategies”. Examples of the coding process are shown in Table 2. The full list of ORRCA domains and examples of codes within each domain can be seen in Supplementary file 1.

**Table 2:** Example of the coding process

**Example from the PIL**	**Pre-determined code**	**ORRCA Domain**
“As a thank you for taking part in the study and for completing the questionnaires, we will reimburse you for your time on this study after you have completed the 6 month questionnaire”.	**Monetary incentive—conditional**: direct cash provided to participants/gift vouchers, prizes that are monetary**—**conditional based on the participant doing/completing an activity.	B2. Monetary incentives
“Although you won’t be paid to take part in the study, we are able to cover all your expenses to attend the study visits. In most cases, we are happy to book a taxi for you to attend and go home afterwards, as well as support any costs for food and drink”.	**Supporting participation**: If participants’ travel fees are remunerated, parking, day-care for their children is covered by the trial.	B7. Supporting participation
“We would be very grateful if you would complete them and post them back in the provided prepaid envelope”.	**Data collection location and method**: If participants are provided with pre-paid return envelopes. If participants are offered multiple/alternative options of data collection.	A3. Data collection location and method

#### The reporting phase

We report the results regarding the communication of retention strategies in terms of SPIRIT items 18b(i) plans to promote participant retention, 18b(ii) plans to complete follow-up including a list of any outcome data to be collected for participants who *discontinue* from intervention protocols and item 18b(iii) plans to complete follow-up including a list of any outcome data to be collected for participants who *deviate* from intervention protocols [27, 28]. We present this information from the PILs, and we then compare the PIL and protocol content.

## Results

**Fig. 1:**
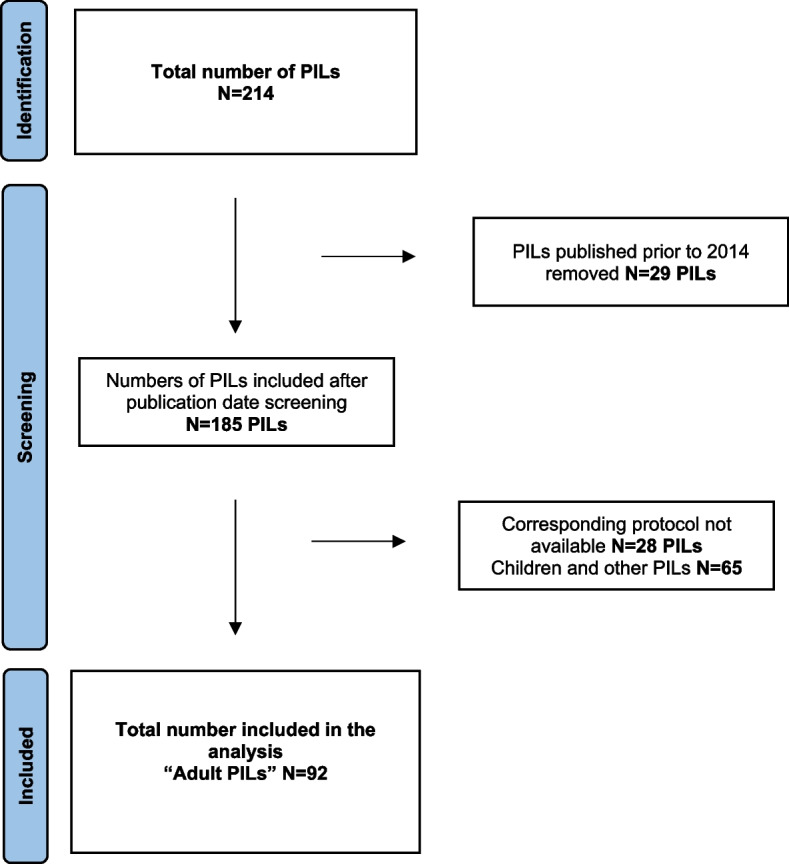
PRISMA flow diagram. Flow diagram showing the number of participant information leaflets at each stage of the screening process

Ninety-two PILs corresponding to 90 RCT protocols were included in the analysis. Figure 1 displays the flow diagram, showing the number of PILs at each stage of the screening process.

### Participant information leaflet characteristics (results in Table 3)

**Table 3:** PIL characteristics

**PIL characteristics**	**Number of PILs (%) Total ** ***N*** ** = 92 PILs from 90 trials thus 90 protocols**
**Year**	**Number of PILs (% out of a total of 92 PILs)**
2014	9 (9.8%)
2015	12 (13%)
2016	19 (20.7%)
2017	15 (16.3%)
2018	22 (23.9%)
2019	3 (3.3%)
2020	7 (7.6%)
2021	4 (4.3%)
Not clear	1 (1.1%)
**Funding status**	
Non-commercial	84 (91.3%)
Commercial^a^	7 (7.6%)
Not clear	1 (1.1%)
Individually randomised trials	88 (95.7%)
Cluster randomised trials	3 (3.3%)
Not clear	1 (1.1%)
**Sample size**	
200 and less	12 (13%)
201–400	14 (15.2%)
401–600	14 (15.2%)
601–800	5 (5.4%)
801–1000	6 (6.5%)
1001 and more	12 (13%)
Not clear	29 (31.5%)
Median sample size	500 participants
**Study population**	
Vulnerable^b^	15 (16.3%)
Mix of vulnerable and non-vulnerable populations	10 (10.9%)
Not vulnerable	2 (2.2%)
Not clear	65 (70.7%)
**Intervention type**	
Non-drug	39 (42.4%)
Drug	31 (33.7%)
Surgical	8 (8.7%)
Mix of the above intervention types	14 (15.2%)
**Mode of follow-up**	
Clinic visit	12 (13%)
Postal follow-up	6 (6.5%)
Use of routine databases/routine data	3 (3.3%)
Data collected via home/site visits	2 (2.2%)
Telephone follow-up	1 (1.1%)
All data collected while participant is in hospital	1 (1.1%)
Online follow-up	1 (1.1%)
Combinations of the above methods of follow-up	64 (69.6%)
Not clear from PIL	2 (2.2%)
**Clinical speciality**	**Number of PILs (% out of a total of 92 PILs)**
Obstetrics and gynaecology	14 (15.2%)
Oncology	13 (14.1%)
Nephrology and urology	12 (13%)
Neurology	9 (9.8%)
Musculoskeletal disorders/illnesses	8 (8.7%)
Cardiology	7 (7.6%)
Vascular diseases	5 (5.4%)
Public health	4 (4.3%)
Infectious disease	3 (3.3%)
Gastroenterology	3 (3.3%)
Respiratory illnesses	2 (2.2%)
Immunology	2 (2.2%)
Ophthalmology	2 (2.2%)
Dermatology	2 (2.2%)
Diabetes and endocrinology	2 (2.2%)
Dental health	1 (1.1%)
Mental health	1 (1.1%)
Genetic conditions	1 (1.1%)
Sexual health	1 (1.1%)

^a^Commerial trials were defined as a trial that has any type of funding or donation from a private for-profit company/organisation for example partly funded by pharmaceutical companies or product provided by a commercial company was classified as a commercial trial

^b^Vulnerable populations were defined via local ethics committee definition [35] and ICH-GCP definition [21]; these included infants and children aged 17 years and under, pregnant women, institutionalised individuals (prisoners, in nursing homes, mental health institutions), critically ill/ICU patients/patients on ventilators unable to provide consent so deferred consent is gained, where stated in the protocol deferred consent is obtained, adults aged 60 and over, participants with learning disabilities, suffers of dementia, adults with terminal illness, homeless individuals and refugees, adults with mental illness, and members of the armed forces and medical/nursing/dental/pharmacy students where there is a hierarchy in the trial that would influence the decision to take part voluntarily

Though we collected PILs from the UK and Ireland, only PILs from the UK remained after the exclusions. In summary, the majority, 95.7% (*n* = 88/92), were for individually randomised trials. PILs for non-drug trials made up 42.4% (*n* = 39/92) and 91.3% (*n* = 84/92) were for non-commercial trials. A full breakdown of the PIL characteristics are presented in Table 3.

### Communication of plans to use strategies to promote participant retention in PILs

#### Proactive plans to use strategies to promote participant retention (results in Table 4)

**Table 4:** Frequency and quotes from the PILs of SPIRIT item 18b(i) plans to promote participant retention

**Does the PIL report SPIRIT item 18b(i) “plans to promote participant retention”**	**Number of PILs (% out of a total of 92 PILs)**
**Yes**	**75 (81.5%)**
No	17 (18.5%)
**Examples from the PILs of plans to use strategies to promote participant retention. Percentage out of the number of PILs with a retention strategy (** ***n*** ** = 75)**	
**“Combined strategies”** 43 (57.3%)	“Filling out the diary will take about five minutes at the end of each day. This can either be on paper form or over the internet. We will send you a brief text message or phone you every week to remind you complete this”. – “Reasonable travel expenses will be reimbursed for all research visits”.
	“We will provide you with a £10 high street voucher each time you complete the package of questionnaires as a thank you. If two parents from each family take part in the study, the second parent will be given a £15 high street voucher as a thank you for each time the questionnaire package is completed” – “You will have the choice to complete these questionnaires yourself by post, or with a researcher during a visit to your home or over the telephone”.
	“We ask that you complete and return this to the Research team in the stamped addressed envelope provided. We will remind you with a letter and via telephone if you do not return your questionnaires”. – “Travel expenses for your research appointments will be refunded by the Research team. We are not able to pay travel expenses for your physiotherapy treatment”.
**Data collection scheduled with routine care** 12 (16%)	“The study visits fit into your normal clinical care”.
	“You will be followed up as you normally would at your hospital so there are no extra visit”.
**Supporting participation** 9 (12%)	“All travel costs will be reimbursed and parking will be provided free of charge. For patients unable to drive, taxi transfers can be provided free of charge to and from the hospital”.
	“You will not be paid for taking part in this trial. However, we will reimburse you for any travel expenses you incur for visits resulting from your participation in the trial, as by participating in the trial, you will be asked to attend more clinics at your GP surgery. If independent travel is difficult for you and might preclude your participation in the trial, you can contact the trial team directly who will discuss alternative arrangements for travel with you”.
**Data collection location and method** 6 (8%)	“You will be asked to complete these, and then post them back to [trial unit] in the freepost envelope they will send you”.
	“Completing 6 short questionnaires on your muscle symptoms every 2 months. There are a few ways in which you can choose to complete the questionnaires; via the web, verbally over the phone, mobile phone app or conventional paper form”.
**Monetary incentives** 3 (4%)	“You will receive £40 in shopping vouchers for completing the study”.
**Reminders** 1 (1.3%)	“The study team will telephone you to remind you to return your questionnaires”.
**Contact information** 1 (1.3%)	“With your permission, we may also contact your General Practitioner (GP) prior to contacting you, or if we are not able to reach you directly”.

SPIRIT item 18b(i) “plans to promote participant retention” were communicated in 81.5% (*n* = 75/92) of PILs. The categories and examples of the strategies to promote participation retention are outlined in Table 4. We found 57.3% (*n* = 43/75) of PILs communicated plans to use “combined strategies” to promote participant retention. The joint most common “combined strategies” were “supporting participation” combined with “data collection location and method”, and “data collection location and method” combined with “data collection scheduled with routine care” (14%;* n* = 6/43). “Data collection location and method” encompasses activities such as travel remuneration, providing pre-paid return envelopes and choice of data collection methods. See a full breakdown of all “combined strategies” in Supplementary file 1.

In terms of individual strategies to promote retention, the most common was telling the participants that data collection for the trial would be scheduled during routine care visits (16%; *n* = 12/75 PILs). Methods of supporting participation such as covering the cost of travel to trial appointments were next most frequently used (12%; *n* = 9/75). The use of reminders and collecting additional contact information were the least popular strategies (1.3%; *n* = 1/75 PIL).

#### SPIRIT item 18b(ii) and item 18b(iii); reactive plans to continue collecting follow-up data for participants who discontinue and deviate from trial protocols

SPIRIT item 18b(ii) “Plans to complete follow-up including lists of any outcome data to be collected for participants who *discontinue* from intervention protocols” ([27]:3) were communicated in 44.6% (*n* = 41/92) of PILs, e.g. “You can withdraw from the study at any time without giving a reason. However, we would like to keep in contact with you to let us know your progress”. SPIRIT item 18b(iii) “plans to complete follow-up including a list of any outcome data to be collected for participants who *deviate f*rom intervention protocols” ([27]:3) were not communicated in any of the PILs.

### The importance of retention in the trial and the impact of missing data (results in Table 5)

**Table 5:** The importance of retention in the trial and the impact of missing data on the ability to answer the research question

**Does the PIL explain the importance of retention in the trial/the importance of continued follow-up in the trial?**	
	**Number of PILs (%)**
**Yes**	**6/92 (6.5%)** **Examples from PILs** “It is really important that you try to complete all follow-up assessments, whichever group you are allocated to, and even if you stop making use of the online sites. This ensures we have all the information we need to properly test how well each site works in improving outcomes for relatives (or close friends)” “For *[trial name]* to produce the best results it is important that participants stay in the study for the entire time.”
No	86/92 (93.5%)
**Does the PIL explain the impact of missing or deleted trial data** ^a^ ** on the ability to answer the clinical question?**	
	**Number of PILs (%)**
**Yes**	**5/92 (5.4%)** **Examples from PILs** “Even if you stop taking part in the study, the information we have recorded about you and the samples you have provided whilst you were in the study may still be used. You can ask for these to be destroyed but please consider, and perhaps discuss with us, how valuable these are to our research before making your decision”. “The questionnaires will be completed when you attend for your treatment or follow-up appointments and will take approximately five minutes to complete. It is very important for you to answer all the questions in the questionnaire for us to accurately assess the impact of the treatment upon you”.
No	87/92 (94.7%)

^a^Deleting participant data collected prior to withdrawal [14]

Quotes relevant to the importance of retention in the trial and the impact of missing data are shown in Table 5. The importance of retention in the trial was explained in 6.5% (*n* = 6/92) of PILs. Explaining the impact that missing or deleted data (deleting data collected prior to withdrawal) has on the ability of the trial to answer the research question [14] was mentioned in 5.4% (*n* = 5/92) of PILs. These explanations focused on explaining that the data collected in the trial is valuable to the research/study.

### Patient and public involvement outlined in the PIL

In terms of patient and public involvement (PPI), 7.6% (*n* = 7/92) of the PILs communicated that there was PPI involvement in the trial. One example is as follows “A group of patients and members of the public helped to develop this research topic and the research questions that should be asked. The group helped to design the study and develop this leaflet. They will continue to be involved throughout the study”. See Supplementary file 1 for more examples.

### Comparison of strategies to promote participant retention in the PILs and their corresponding protocols (results in Table 6)

**Table 6:** PIL content compared to protocol content; plans to use strategies to promote participant retention

**Communication the same information**	
**PIL content**	**Protocol content**
We will send you up to two reminders and will aim to contact you by post, email and/or telephone, taking into account which communication method is best for yourself	At 6 weeks after surgery, participants will complete a questionnaire to measure Pain Numerical Rating Scales (NRS), time to return to normal activities and acceptability, EQ5D and SF12. At 6 months after surgery and at 15 months following randomisation, participants will complete the SF12, MMAS, EQ5D, satisfaction with treatment and questions about healthcare utilisation. Participants will receive up to two reminders by post, email or phone, taking into account any preferences they may have for mode of communication
These follow up appointments will coincide with your usual regular clinic appointments	Patients will be assessed at 3 monthly intervals from baseline to 3 years in the patient’s routine outpatient clinic visit as per the trial schedule of assessments
**Partially communicate the same information**	
**PIL content**	**Protocol content**
We will ask you for your name, email address and alternative telephone numbers so that we can contact you to find a suitable time to conduct these questionnaires. In the event that we are unable to contact you, for example your contact details change, we may request further information about you from your Local Authority so that we can reach you - For your time and contribution to the study we would like to provide you with a voucher worth £20 for each questionnaire you take part in as a thank you for taking part	We will make every effort to ensure retention. At enrolment, participants will be asked to provide alternative telephone numbers, email addresses and any other forms of communication that may be helpful to contact them. Researchers will endeavour to build a positive rapport with each participant for subsequent follow-up. Participants will also be emailed/posted vouchers as a reimbursement for their time after each questionnaire. **Finally, a third-party text messaging platform (Esendex) will be used to send text messages to participants to keep in touch, or remind them of their follow-up contact**
We will provide you with a £10 high street voucher each time you complete the package of questionnaires as a thank you. If two parents from each family take part in the study, the second parent will be given a £15 high street voucher as a thank you for each time the questionnaire package is completed - You will have the choice to complete these questionnaires yourself by post, or with a researcher during a visit to your home or over the telephone	Retention strategy To maintain engagement, encourage retention and to thank family caregivers for their time, primary carers will be provided with a £10 high street voucher when contacted to complete follow-up data collection, as has previously been shown to be effective. **Contact details will be collected during recruitment, and participants will be reminded by email and text message when a data collection follow-up is due and to complete questionnaires when posted. Participants will also receive a study newsletter at approximately 9–10 months post-randomisation to maintain participant engagement**. Participants will be offered three methods of data collection: via telephone, postal or face-to-face at a convenient location. **For non-responding participants, a minimum data set (consisting of 3 prioritised outcome measures (Warwick Edinburgh Well-Being Scale, EQ-5D and Parenting Sense of Competence Scale) aligning with the intervention logic model and taking into consideration participant burden) will be offered to reduce participant burden and maximise follow-up rates.**
**Do not communicate the same information**	
**PIL content**	**Protocol content**
We will offer you a total of £20 in vouchers as a thank you for taking part. We will offer £10 for completing the first set of questionnaires and another £10 when you complete the third set of questionnaires at 12 months - Initial postal questionnaire sent with a pre-paid return envelope	All participants will be provided with study progress updates at 3 and 9 months via a newsletter to maintain engagement with the trial and encourage response rates of follow-up questionnaires. The newsletter does not provide any detail on the *[study name]* intervention—All participants complete self-reported outcome measures in the form of questionnaires (IPSS, ICIQ-UI-SF,EQ-5D-5 L and B-IPQ) at baseline (postal) and 6 and 12 months (postal, online or phone) post-enrolment. Participants are sent one reminder to return their baseline materials, and up to three reminders to return their 6- and 12-month questionnaires.
You will not be paid for taking part in this trial. However, we will reimburse you for any travel expenses you incur for visits resulting from your participation in the trial, as by participating in the trial, you will be asked to attend more clinics at your GP surgery. If independent travel is difficult for you and might preclude your participation in the trial, you can contact the trial team directly who will discuss alternative arrangements for travel with you	Where questionnaires are not validated for use on a tablet computer, or where individuals are not comfort able using one, paper copies will be made available for completion
**Examples of plans to use strategies to promote participant retention communicated in the PILs but not outlined in the trial protocol**	
“All travel costs will be reimbursed and parking will be provided free of charge. For patients unable to drive, taxi transfers can be provided free of charge to and from the hospital”	
“To collect the information we need, everyone in the study will be sent questionnaires by post approximately 1, 3, 6, 18 and 27 months after you join the study. The questionnaires ask about your vision and general health. We will send you up to two reminders and will aim to contact you by post, email and/or telephone, taking into account which communication method is best for you”	

Overall, 81.5% (*n* = 75/92) of PILs report “plans to promote participant retention”; these 75 PILs correspond to 74 protocols (two PILs correspond to one protocol). Of these 75 PILs and 74 corresponding protocols, there are 59 PILs corresponding to 58 protocols where both the PIL and the protocol communicate plans to use strategies to promote participant retention.

Out of the 59 PILs and 58 matching protocols, 18.6% (*n* = 11/59) communicated the same information relevant to the participant, and the remaining 81.4% (*n* = 48/59) of PILs either only partially communicated (45.8%; *n* = 27/59) the same information or did not communicate the same information (35.6%; *n* = 21/59) as the protocol regarding the retention strategy(ies). In 21.3% of PILs (*n* = 16/75) that outlined a plan to promote participant retention, this plan was not communicated in the corresponding protocol. Examples are shown in Table 6. A full list of all protocol and corresponding PIL content is in Supplementary file 1.

## Discussion

This study has demonstrated that a high proportion of PILs outlined plans to use strategies to promote participant retention (81.5%; *n* = 75/92) but not all corresponding protocols outlined or transparently communicated plans to use strategies to promote retention. Information was either partially communicated or completely different information was communicated in the PIL when compared to the protocol. Among PILs and corresponding protocols that both outlined a retention strategy(ies), there was a miscommunication regarding the retention strategy(ies) in 81.4% (*n* = 48/59 PILs) of PILs compared to their corresponding protocol. Not all participant relevant information about plans to use retention strategies outlined in the protocol were communicated in the PIL, for example participants were not informed about reminders, newsletters, or the option for paper-based questionnaire completion via PILs despite these plans being outlined in the corresponding protocol (see Table 6 and Supplementary file 1 for more examples). Miscommunication may potentially cause issues, if participants are not aware and do not expect to receive, for example, reminders if they miss data collection points. This could negatively impact their feelings towards remaining in the trial and lead to drop out. Poor trial expectation setting may be a reason why participants no longer remain in trials [19]. Prior research shows that among participants who failed to return trial questionnaires at least once, the trial activities did not meet their initial expectations. They did not expect to receive the same questionnaire more than once and therefore did not send it back [20]. Trial expectation setting should involve clearly communicating information regarding retention strategies that will be used in the trial via PILs. We have shown there is a lack of follow-through when communicating this information in PILs. It is unclear why this is the case. Trial teams may fear that providing too much information may deter participation and negatively impact recruitment. Conversely though, improving knowledge and setting expectations regarding retention may lead to more meaningful participation and improve retention, but this is only speculation [17].

Using newsletters, pre-paid envelopes for returning questionnaires, telephone reminders and collecting data during routine care visits are all within the top 10 most routinely used retention strategies in the UK [13]. This finding is reflected in the PILs, e.g. the most popular individual retention strategy was “data collection scheduled with routine care” (16%; *n* = 12/75). These routinely used retention strategies are also seen in the “combined strategies”. There are signs that trial teams are planning to tackle the issue of poor retention during the early stages of the trial and communicating these plans with potential trial participants during recruitment, as evidenced by the inclusion of retention strategies in protocols from previous work [26] and protocols and PILs in this work. There is, however, no high-quality supporting evidence to show that the majority of retention strategies that are being used in trials improve participant retention rates [3]. Based on cost estimates, these strategies can be very expensive to implement [36] and the cost-effectiveness of retention strategies is yet to be shown [37]. Early planning of strategies (ideally during grant application and subsequent protocol development) to improve retention may increase the likelihood of successful implementation of these plans. Certainly, if planned for early, the resources that are needed will be budgeted from the outset. Without supporting evidence however, resources are still potentially being wasted implementing activities that may or may not improve participant retention rates.

In 21.3% (*n* = 16/75) of PILs that communicated plans to use retention strategies to promote participant retention, these retention strategy plans were not included in the corresponding trial protocols. This would indicate that although trial teams plan to use retention strategies during the planning stage of the trial, they do not communicate this information in the trial protocols. We know very little as to why this is the case, and we plan to do a follow-up qualitative interview study with members of trial teams to tease this out. We know that the SPIRIT 2013 guidelines recommend the inclusion of plans to promote participant retention [27, 28] but do not know why trial teams fail to do so. Our findings here lead us to believe it is a reporting issue rather than a planning issue. This poses issues for the replication of plans to promote participant retention, which is needed to produce evidence to evaluate the effectiveness of the retention strategy in the future. Evaluating retention strategies is essential considering the lack of existing evidence to support their use in trials [3]. While it is encouraging that trial teams are considering retention during the early stages of trial development and communicating plans to use strategies to promote retention, reporting it in the protocol is essential for good practice.

Although trial teams are reasonably good (81.5% of PILs; *n* = 75/92) at informing potential trial participants about their intention to promote participant retention, the value and importance of ongoing participation to the trial results are not well communicated in PILs (6.5%; *n* = 6/92). This is a well-known phenomenon, whereby the potential harms are well communicated but potential benefits receive less attention [38]. Even less information is provided on the impact that missing or deleted (deleting data collected prior to withdrawal) data has on the ability of the trial to answer the research question in the PILs (5.4%;* n* = 5/92). These findings are reflective of previous research [14, 15]. Without providing information regarding the importance of retention/the consequences of leaving for the trial, participants may be unaware of the value of their ongoing participation. Interviews with trial participants who had discontinued trial participation by either not returning for at least one follow-up clinic visit or not returning at least one follow-up questionnaire found some participants could not identify the negative consequences of not returning follow-up data. A reason for this cited by authors was due to the fact participants expected the trial team to send another questionnaire or reschedule the appointment, further adding to the need for clearer communication regarding what is involved in trial follow-up and ongoing participation requirements. Additionally, some participants were unsure their contributions made much of a difference to the trial [17]. Other research has found that sometimes participants are not aware they are considered non-retainers by trial teams [17, 19]. This raises questions about how much trial participants understand about the requirements for and value of remaining in the trial. It may be valuable to retention rates to explain this information in PILs; potential participants will be better informed about the importance of continuing to provide follow-up data to the trial. Granted, providing information on the value and importance of remaining in the trial may be difficult as it may be perceived as coercive [14] but a balance needs to be achieved between information on withdrawal and the importance of retention, as participants want to know what is expected of them when they participate in a trial [39].

### Strengths and limitations

It is a significant strength in this study that we compared the content regarding plans to use strategies to promote participant retention communicated in the PILs with the corresponding protocol as it allowed for a more detailed analysis of the communication process between trial teams and potential trial participants. We also recognise some weaknesses. Although we aimed to include PILs from both Ireland and the UK, our eligibility criteria meant only PILs and protocols from trial units in the UK were included. However, the sample is representative of PILs from the UK, as it includes PILs from more than 20 CTUs and a variety of populations, clinical specialities and intervention types. Additionally, the findings of this piece of work may not be generalisable to PILs for children and the corresponding parent PILs, PILs for legal/personal representatives and PILs for adults in research without prior consent, as these PILs have not been included in the sample.

PILs are only one source of information that is provided to potential trial participants during the informed consent stage. We are aware that participants may be verbally informed about plans to use strategies to promote participant retention during conversations with trial staff during the consent stage of the trial. This information has not been captured. However, since retention is seldom discussed in consent consultations [15], it is likely that plans to use strategies to promote participant retention are also seldom mentioned.

### Recommendations

We recommend and advocate for PILs to be transparent and include information specifically regarding retention strategies, and information on the value and importance of remaining in a clinical trial, as we feel it is important to set trial expectations with the hope of leading to improved retention rates. We acknowledge, however, that existing PILs are exceptionally long and that a balance needs to be struck between providing potential participants with information on retention and the risk of information overload. Potential trial participants have outlined that they want to know what is expected of them if they take part in a clinical trial, and this includes information on follow-up and retention requirements [39]. Additionally, most people only access the minimum required information that provides a broad understanding of a project and what would be required if they chose to participate [40]; this again speaks to retention. Trial teams may not want to outline certain retention strategies such as the option of collecting the minimum dataset needed for the primary outcome, as it may mislead participants to what is expected in terms of ongoing trial participation. It is understandable why this information is not included in the PIL but in these scenarios, we advocate for participation to be made as burden-free as possible from the outset of the trial. For retention strategies such as reminders, prompts, incentives and other activities that participants may be receiving, we recommend trial teams to outline these from the outset of the trial. Additionally, it is also recommended that trial teams make it clear to participants if trial withdrawal can be nuanced. Depending on the trial, participants may be able to continue participating with less commitment if they are struggling to meet all trial requirements, or if they decide to stop receiving the intervention, so that their follow-up data can still be collected and valuable to the trial results [41].

Despite the communication of plans to use retention strategies to promote retention in the PILs, there is still a lack of evidence to support the use of many strategies that trial teams are planning [3]. We recommend that if trial teams are planning on using strategies to promote retention that they also plan to evaluate these strategies to add to the evidence base to either support or not support the use of retention strategies going forward. Trialist can evaluate retention strategies by using a SWAT (study within a trial) on which there is lots of guidance [42] and the Northern Ireland SWAT repository (SWAT Store | The Northern Ireland Network for Trials Methodology Research (qub.ac.uk)) contains protocols for SWATS that are focused on improving retention in trials that need to be evaluated in order to determine if they are effective or not at improving retention rates. We encourage trialists to look at these resources if they are interested in formally evaluating retention strategies. Clear communication of plans to use retention strategies in the trial protocol will also allow for replication of these methods for future evaluations.

We recommend that when choosing a retention strategy, the suitability of the strategy to the target population should also be considered. Similar to the findings of our recent scoping review [26], among the PILs, most retention strategies were generic retention strategies that did not specifically target groups more “at risk” of dropping out, for example participants who actively withdraw from the trial. Research using individual patient data within specific contexts has been able to identify participants most at risk of leaving the trial, for example participants with co-morbidities [43], these groups may need to be specifically targeted going forward depending on clinical context. Participant demographic characteristics may need to be considered by trial teams going forward as there may also be cultural and generational differences in terms of preferences for retention strategies and what different groups find acceptable. Having PPI input on retention strategies may help with this decision, although evidence as to whether PPI aids in participant retention is uncertain [44]. Irrespective of this, participants want to know how patients and members of the public have been involved in the design of the study [39]. Perhaps, going forward this is a piece of information that trial teams should try to include in PILs.

## Conclusion

Retention strategies are often communicated in PILs, but the information provided to the potential trial participant is different from the information outlined in the protocol. This miscommunication may negatively impact participants’ trial expectations. Furthermore, without explaining the importance of retention in the trial, it is hard to expect potential participants to understand the reasons why these activities are conducted. Providing this information sets trial expectations and provides participants with a more well-rounded understanding of participation and withdrawal in a trial. Additionally, in some cases, plans to promote retention outlined in the PIL are not included at all in the corresponding protocol. While results indicate that trial teams appear to be considering participant retention at the early stages of the trial, they do not always communicate this information in the trial protocol nor communicate the same information in the PIL as outlined in the protocol.

### Supplementary Information

Supplementary Material 1.

## Data Availability

The dataset created, used and analysed during this project is available from the authors on reasonable request.

## Abbreviations

RCTs: Randomised controlled trials
PILs: Participant information leaflets
CTUs: Clinical Trial Units
SPIRIT: Standard Protocol Items: Recommendations for Interventional Trials
PPI: Patient and public involvement
ORRCA: Online Resource for Research in Clinical Trials
SWAT: Study within a trial
