# Supplementary material for: Retention strategies are routinely communicated to potential trial participants but often differ from what was planned in the trial protocol: an analysis of adult participant information leaflets and their corresponding protocols
Source: Trials. 2024 Jun 10;25:372. doi: 10.1186/s13063-024-08194-7 (PMC11163762; doi:10.1186/s13063-024-08194-7)
Supplement: Supplementary file 1 — Supplementary Material 1. [file 13063_2024_8194_MOESM1_ESM.docx]

**Supplementary File 1.**

**Table of Contents Page Number**

**1.** Examples of codes mapped to the ORRCA retention domains **3**

**2.** **“Adult PILs” Analysis results**

2.1 List of all ‘combined (retention) strategies’ **4**

2.2 The reporting of SPIRIT guidelines and SPIRIT item 18b in the corresponding trial protocols **5**

2.3 Comparisons of PIL versus protocol content of plans to use strategies to promote participant retention **6**

2.4 Mention of Patient and Public Involvement in the PILs **23**

Abbreviations and Reference **23**

1. **Examples of codes mapped to the ORRCA retention domains**

Codes that were used based on the existing literature of retention strategies identified by the Cochrane Review on strategies to improve retention in randomised trials (1) and retention strategies that were identified and routinely used by UK Clinical Trial Units (CTUs) (2). These codes were then mapped to the ORRCA (Online Resources for Research in Clinical triAls) retention domains (3). Where multiple codes were identified within a single participant information leaflet a new code was developed; ‘combined strategies’. Within this new code the individual codes were also identified.

We mapped strategies based on the groupings outlined by ORRCA, examples shown in table 1.

| **Table 1: Codes mapped to ORRCA** | |
| --- | --- |
| **Retention Domains**  **ORRCA retention domains** | **Retention strategies we placed into each group** |
| **A. Data collection** |  |
| **A3. Data collection location and method** | If participants are provided with pre-paid return envelopes  If participants are offered multiple/alternative options of data collection |
| **A5. Data collection during routine care** | If data collection was during a routine clinic visit |
|  |  |
| **B. Participants** |  |
| **B1. Reminders**  **Prompts** | If participants are sent any type of reminder (phone call, text, email, postal) for when they miss a data collection point.  If participants are sent any type of prompt (phone call, text, email, postal) for upcoming data collection points |
| **B2. Monetary incentives – direct cash provided to participants/gift vouchers, prizes that are monetary**  **Conditional Incentives**  **Unconditional incentives** | If participants are offered direct cash payments for data collection points, gift vouchers, prizes that are monetary, entry to raffles for monetary prizes.  Conditional – if they have to complete a task to obtain the incentive.  Unconditional – they receive the incentive regardless of completing the activity |
| **B3. Non-monetary incentives –entry to raffles for prizes that are non-monetary, completion of trial certificates, offering the controls the intervention at the end of the trial**  **Conditional incentives**  **Unconditional Incentives** | If participants are offered any type of non-monetary incentive, completion of trial certificates, offering the controls the intervention at the end of the trial.  Conditional – if they have to complete a task to obtain the incentive.  Unconditional – they receive the incentive regardless of completing the activity |
| **B4. Maintaining participant engagement** | If participants are sent newsletters during the trial to keep them updated on trial activity, if participants are sent |
| **B7. Supporting participation** | If participants travel fees are remunerated, parking, day-care for their children is covered by the trial |
| **B8. Contact information** | If the trial collects multiple forms of contact for the participant, alternative contact person for the participant |
|  |  |
| **Combined strategies** | If is where any of the above strategies are used in combination with each other within the trial, we coded them as “combined methods” – the individuals strategies were also identified. |

1. **“Adult PILs” analysis**

**2.1 List of all combinations of retention strategies**

Fifty-seven percent of (n=43/75) participant information leaflets (PILs) communicated plans to use ‘combined strategies’ to promote participant retention. Table 2 shows all combinations of strategies planned to promote retention.


| **Table 2: All combinations of plans to use strategies to promote participant retention** | |
| --- | --- |
| **‘Combined strategies’** | **Number of PILs (total n=43)** |
| Data collection location and method  Data collection scheduled with routine care | 6 |
| Supporting participation  Data collection location and method | 6 |
| Reminder  Data collection location and method | 5 |
| Monetary incentive – conditional  Data collection location and method | 3 |
| Reminder  Supporting participation  Data collection location and method | 3 |
| Reminder  Data collection location and method  Data collection scheduled with routine care | 2 |
| Reminder  Monetary incentive - conditional  Data collection location and method | 2 |
| Supporting participation  Data collection scheduled with routine care | 2 |
| Reminder  Supporting participation  Data collection location and method  Data collection scheduled with routine care | 2 |
| Prompt  Reminder  Supporting participation  Data collection location and method  Data collection scheduled with routine care | 1 |
| Contact information  Monetary incentive - conditional | 1 |
| Reminder  Contact information  Data collection location and method  Data collection scheduled with routine care | 1 |
| Supporting participation  Monetary incentive – conditional | 1 |
| Monetary incentive – unconditional  Data collection location and method | 1 |
| Monetary/Non-monetary incentive – conditional  Data collection location and method | 1 |
| Reminder  Data collection location and method  Monetary incentive – conditional and unconditional  Data collection scheduled with routine care  Maintaining staff engagement | 1 |
| Reminder  Data collection location and method  Monetary/Non-monetary incentives – conditional | 1 |
| Reminder  Contact information | 1 |
| Monetary incentives – conditional  Supporting participation  Data collection location and method | 1 |
| Supporting participation  Data collection location and method  Data collection scheduled with routine care | 1 |
| Supporting participation  Monetary incentives conditional  Data collection location and method | 1 |

**2.2 *The reporting of SPIRIT guidelines and SPIRIT item 18b in the corresponding trial protocols***

We report the results regarding the communication of retention strategies in terms of SPIRIT items 18b(i) plans to promote participant retention, 18b(ii) plans to complete follow-up including list of any outcome data to be collected for participants who *discontinue* from intervention protocols, and item 18b(iii) plans to complete follow-up including list of any outcome data to be collected for participants who *deviate* from intervention protocols (4, 5).

Of the 90 corresponding trial protocols, 35.6% (n=32/90) of protocols reported using the SPIRIT 2013 guidelines when developing the protocol. Of the protocols that reported using the SPIRIT 2013 guidelines 81.3% (n=26/32) reported item 18b(i) “plans to promote participant retention”. Reporting of “plans to complete follow up including list of any outcome data to be collected for participants who *discontinue* (item 18b(ii)) or *deviate* (item 18b(iii)) from intervention protocols” (5:3) were reported in 25% (n=8/32) and 6.3% (n=2/32) of protocols respectively.

Regardless of using the SPIRIT guidelines, protocols still reported retention strategies. Of the 90 trial protocols in total, 74.4% (n=67/90) reported plans to use strategies to promote participants retention.

Table 3 provides a full breakdown of information regarding the protocols included in the analysis and whether they reported SPIRIT item 18b.

| **Table 3: Corresponding protocol information on plans to use retention strategies according to SPIRIT (4)** | |  |
| --- | --- | --- |
| **Publication location** | **Number of protocols (% out of 90 protocols)** |  |
| Protocols published in a peer reviewed journal | 73/90 trial protocols (81.1%) |  |
| Protocols published outside a peer reviewed journal e.g. trial website | 17/90 trial protocols (18.9%) |  |
|  |  |  |
| **Number of corresponding protocols that reported using the SPIRIT 2013 guidelines** |  |  |
| Yes | 32/90 trial protocols (35.6%) |  |
| No | 58/90 trial protocols (64.4%) |  |
|  |  |  |
| **Reported using SPIRIT and reported item 18b(i) “plans to promote participant retention”** |  |  |
| Yes | 26/32 protocols (81.3%) | These 26 protocols have 27 corresponding PILs of which 22 PILs also report 18b(i) (81.5%) |
| No | 6/32 protocols (18.8%) |  |
|  |  |  |
| **Reported using SPIRIT and reported item 18b(ii)** |  |  |
| Yes | 8/32 protocols (25%) | These 8 protocols have 9 corresponding PILs of which 2 report item 18b(ii) (22.2%) |
| No | 24/32 protocols (75%) |  |
|  |  |  |
| **Reported using SPIRIT and reported item 18b(iii)** |  |  |
| Yes | 2/32 protocols (6.3%) | These 2 protocols have 3 corresponding PILs of which 0 report item 18b(iii) (0%) |
| No | 30/32 protocols (93.8%) |  |
|  |  |  |
| **Corresponding protocols that reported 18b(i) “plans to promote participant retention” regardless of reporting the SPIRIT guidelines** |  |  |
| Yes | 67/90 protocols (74.4%) | These 67 protocols correspond to 69 PILs of which 59 PILs reported 18b(i) (85.5%) |
| No | 23/90 protocols (25.6%) |  |
|  |  |  |
| **Protocols that reported item 18b(ii) regardless of reporting the SPIRIT guidelines** |  |  |
| Yes | 28/90 protocols (31.1%) | These 28 protocols correspond to 30 PILs of which 15 PILs reported 18b(ii) (50%) |
| No | 62/90 protocols (68.9%) |  |
|  |  |  |
| **Protocols that reported item 18b(iii) regardless of reporting the SPIRIT guidelines** |  |  |
| Yes | 4/90 protocols (4.4%) | These 4 protocols correspond to 5 PILs of which 0 reported 18b(iii) (0%) |
| No | 86/90 protocols (95.5%) |  |
|  |  |  |
| **Protocols that report PPI involvement** |  |  |
| Yes | 49/90 protocols (54.4%) | These 49 protocols have 50 corresponding PILs of which 5 report PPI (10%) |
| No | 41/90 protocols (45.6%) |  |

**2.3 Comparison of PIL content versus protocol content in terms of communicating plans to use strategies to promote participant retention**

Overall, 81.5% (n=75/92) of PILs report “plans to promote participant retention”, these 75 PILs correspond to 74 protocols (two PILs correspond to one protocol). Of these 75 PILs and 74 corresponding protocols, there are 59 PILs corresponding to 58 protocols where both the PIL and the protocol communicate plans to use strategies to promote participant retention. Table 4 shows all the plans to use strategies to promote retention in the protocols and their corresponding PILs.

| **Table 4: Communicate the same information regarding plans to use a strategy to promote participant retention** | |
| --- | --- |
| **PIL content** | **Protocol content** |
| These follow up appointments will coincide with your usual regular clinic appointments | Patients will be assessed at 3 monthly intervals from baseline to 3 years in the patient’s routine outpatient clinic visit as per the trial schedule of assessments |
| If you agree to take part in the trial, your hospital visits will be at the times when you would normally have hospital visits for your burn injury - There will be no additional hospital visits to make for the trial and all hospital visits will be at times when you would normally have appointments for your burn injury, as such, there will be no provision for travel expenses) | The fit and wear of the garments was checked throughout the trial at the routine clinic visits and garments were replaced as required.  All participants were required to attend routine follow-up scheduled appointments |
| As part of your standard treatment for ITP, you will have regular blood tests and doctor or nurse reviews to check how you are getting on.  This will be the same whether you chose to take part in this trial or not. There will be no extra doctor visits for the research trial and your usual visits will be used to collect information on how you are | Laboratory and clinical data will be collected  from routine appointments |
| These visits can take place by telephone in order to minimise the number of visits needed to hospital, but you may be asked to attend in person. - You will also be asked to complete questionnaires about your health at the 6,12 and 24 month visits. As above, these may  be done by telephone | This is standard follow up of these patients. In  cases where it is felt to be a risk to bring patients to hospital for follow-up visits, you may conduct these visits by telephone; |
| We will send you up to two reminders and will aim to contact you by post, email and/or telephone, taking into account which communication method is best for yourself | At six weeks after surgery, participants will complete a questionnaire to measure Pain Numerical Rating Scales (NRS), time to return to normal activities and acceptability, EQ5D and SF12. At six months after surgery and at 15 months following randomisation, participants will complete the SF12, MMAS, EQ5D, satisfaction with treatment and questions about healthcare utilisation. Participants will receive up to two reminders by post, email or phone, taking into account any preferences they may have for mode of communication |
| We will also ask you to fill in some questionnaires about your experience either by phone or by post - We will either see you at one of your routine clinic appointments or telephone you to see how you are. | Study visits could be face to face or by phone or other means of communication, for example, via letter or email |
| If for any reason we were unable to follow up with you in clinic we may contact you by telephone to as about your wellbeing since you joined the study - We will do the study follow up within your normal NHS care | The 6, 12, 24, 36, 48 and 60-month follow-up is in person or over the telephone, according to local practice. If contact cannot be made with the participant then medical notes can be used |
| Any additional appointments at the hospital that you need to attend because of the study, for example–attending for extra out-patient appointments, you will be able to claim travelling expenses from the research team. -We will try to collect the study information when you come to your psoriatic arthritis clinic visits | Reasonable travel expenses for any visits additional to normal care will be reimbursed on production of receipts, or a mileage allowance provided as appropriate |
| the stitch is normally removed around 38 weeks of pregnancy or when labour starts | Plans to promote participant retention and complete follow-up: The trials team ensures that all outstanding data is queried monthly with all sites. Where babies are not delivered at the site in which the cerclage was performed, follow-up is facilitated through a continuing care site setup processes. - The trial is pragmatic in nature and requires no additional visits or assessments. |
| We may send you up to two reminders and will aim to contact you by post, email,  telephone and/or text message, taking into account which communication method is best for yourself - On return of your 12 week questionnaire and again after the return of your final questionnaire (sent 12 months after you join the study) we will send you a love2shop high street voucher to thank you for completing the questionnaires. Please let us know if you do not wish to receive these vouchers - | Reminders may be used; for the earlier time points this may be a text message or email on the day that the questionnaire is due and, for the later time points (e.g. 12 weeks and 12 months), this reminder will be sent approximately 2 weeks and 4 weeks after the questionnaire is due. We will offer and use all methods of delivery and col lection of questionnaires and reminders including use of research teams for time points associated with hospitalisation, postal mail, email, web-based, telephone and SMS text, taking into account each participant’s stated preferred means of receiving and completing the measures. Participants will be sent a voucher (of modest value) as a token of appreciation for completion and return of the questionnaire - We intend to maintain interest in the trial by publication of *[trial name]* newsletters at intervals for staff and collaborators  [two PILs share this one protocol] |
| We may send you up to two reminders and will aim to contact you by post, email, telephone and/or text message, taking into account which communication method is best for yourself - On return of your  12 week questionnaire and again after the return of your final questionnaire (sent 12 months after you join the study) we will send you a token of appreciation for your time spent on completing the questionnaires. Please let us know if you do not wish to receive these vouchers |  |
| **Partially communicate the same information regarding plans to use a strategy promote participant retention** | |
| **PIL content** | **Protocol content** |
| Please tell use if you would prefer to be contacted by phone, email or post -This study will fit into your usual care. | The participant will be asked to complete the questionnaires during the clinic visit (prior to other clinical assessments) or at home where required - **For forms completed at home, the patient will be provided with a pre-paid, self-addressed envelope which will be used to send the completed questionnaires to the [trial name] trial office*.*** Alternatively women may return completed forms to the [trial name] email account or, for the CSQ 8, **women may complete this electronically via SmartSurvey**. - **To reduce loss to follow-up, the local research team will record the NHS numbers of participants, to trace the participants via local GP practices and non-participating NHS trusts where necessary** - the trial is designed to fit in with routine hospital practice as far as possible, imposing minimal additional workload by keeping extra clinic-based tests and evaluations to a minimum. |
| We will collect most of our study information about your pregnancy and your baby from your hospital notes but we may need to contact you to check some details. Please tell us if you would prefer to be contacted by phone or email - This study will fit into your usual antenatal care, so that you will not have to make any extra hospital visit | Loss to Follow-Up  **To reduce loss to follow-up, the local research team will record the NHS numbers of participants, to trace the participants via local GP practices** - The trial pathway fits within the current standard care pathway for women presenting with early pregnancy bleeding |
| We will post the paper questionnaire out to you about 1 week before your appointment so you can complete it at home and bring it with you to clinic .**Occasionally, we may contact you over the phone** - **For the intervention group only - You will receive reminders when your ePROM report is due. The reminders will be sent by text or email, depending on your preference**. - ALL participants **- We will reimburse your travel expenses (including car parking fees) for all study clinic visits** - Whichever group you are assigned to, you will be closely monitored for the study by the kidney research team at the Queen Elizabeth Hospital every 3 months for 1 year. We would try to do this while you are already in clinic for your routine CKD appointment | This questionnaire may be posted out to participants prior to their scheduled clinic/research visit, but research staff will be on hand in clinic to assist with completion where required. - Since these measures  are routinely collected for clinical monitoring, the results closest to the calculated visit due date will be used for trial data, rather than repeating tests which have already  been performed - very attempt will be made to collect full follow-up data on all study participants; it is thus anticipated that  missing data will be minimal |
| As a thank you for taking part in the study and for completing the questionnaires, we will reimburse you for your time on this study after you have completed the 6 month questionnaire | **To try to maximise follow-up rates, data will**  **be triangulated with routinely collected data on initiation and feeding status at 6–8 weeks and women will be offered a £25 ‘thank you’ voucher if they complete all the follow-ups** |
| We will ask you for your name, email address and alternative telephone numbers so that we can contact you to find a suitable time to conduct these questionnaires. In the event that we are unable to contact you, for example your contact details change, we may request further information about you from your Local Authority so that we can reach you - For your time and contribution to the study we would like to provide you with a **voucher worth £20** for each questionnaire you take part in as a thank you for taking part | We will make every effort to ensure retention. At enrolment, participants will be asked to provide alternative telephone numbers, email addresses and any other forms of communication that may be helpful to contact them. Researchers will endeavour to build a positive rapport with each participant for subsequent follow-up. Participants will also be emailed/posted vouchers as a reimbursement for their time after each questionnaire. **Finally, a third-party text messaging platform (Esendex) will be used to send text messages to participants to keep in touch, or remind them of their follow-up contact** |
| To remind you to complete the questionnaires, we will be collecting your contact details including your name, email address, telephone and mobile numbers, and address so that we can send you a link to the questionnaire either by email or text message. We may also send out a letter asking about your recovery if we can’t get a hold of you or your study partner. We may also call you to ask you to complete it over the phone - We will also be asking someone close to you to act as a study partner. We will collect their contact details including their name, email address and telephone number and with your permission we will contact them in the event that we are not able to contact you after you have been discharged from hospital. We may also ask them to complete the GOSE questionnaire, on your behalf if you are unable to. - The study will not require additional hospital visits over and above the standard of care. | Lost to Follow Up: Sites will contact patients for each visit in accordance with the procedure outlined in section 13.2. If patients are not able to be contacted after three missed regular (fortnightly or monthly) GOSE assessment, a letter will be sent out to the participant asking them to get in contact with the trial team. Participants will not be contacted again until the 3-month, 12 month, or annual follow up occurs. Sites will record all details regarding attempts to contact the participant on the appropriate CRF. To minimise missing data, participants will be able to complete the primary outcome online or over the phone. In addition, contact details for participant’s study partner will be collected and they will be able to complete the primary outcome on behalf of the participant (Patients will have the option of completing the GOSE online, or via the telephone - **Automated alerts will be set up to help patients complete the GOSE in the right time window**, and to inform the study team if this appears not to be happening. -**Once patients are discharged from hospital, they and their study partner will receive a reminder on the day before the GOSE follow up window starts and a reminder on the first day of the follow up window, that the GOSE should be completed** - If the GOSE is not completed on the correct day, the trial team will receive a notification and they will attempt to contact the participant by phone for 4 days (for fortnightly assessments) or 6 days (for monthly assessments), or until the assessment is completed |
| We will provide you with a £10 high street voucher each time you complete the package of questionnaires as a thank you. If two  parents from each family take part in the study, the second parent will be given a £15 high street voucher as a thank you for each time the questionnaire package is completed - You will have the choice to complete these questionnaires yourself by post, or with a researcher during a visit to your home or over the telephone | Retention strategy  To maintain engagement, encourage retention and to thank family caregivers for their time, primary carers will be provided with a £10 high street voucher when contacted to complete follow-up data collection, as has previously been shown to be effective [21]. **Contact details will be collected during recruitment, and participants will be reminded by email and text message when a data collection follow-up is due and to complete questionnaires when posted. Participants will also receive a study newsletter at approximately 9–10 months post randomisation to maintain participant engagement.** Participants will be offered three methods of data collection: via telephone, postal or face-to-face at a convenient location. **For non-responding participants, a minimum data set (consisting of 3 prioritised outcome measures (Warwick Edinburgh Well-Being Scale, EQ-5D and Parenting Sense of Competence Scale) aligning with the**  **intervention logic model and taking into consideration participant burden) will be offered to reduce participant burden and maximise follow-up rates.** |
| The study team will telephone you to remind you to return your questionnaires. | In order to facilitate the process of patient recruitment and data collection into busy routine clinics, data collection can be conducted by any suitably trained clinician  (nurse, health care assistant or GP) - Participants will be telephoned by the trial team 1 week after the due date to remind them to return the questionnaire by post **or to offer to complete these instruments over the telephone at that point. If the questionnaire is not received within 1 week of the first telephone reminder, the trial team will telephone the participant again** |
| However, we will provide you with a £10 high street voucher each time you complete the 3 and 12 month follow-up questionnaires as a thank you everyone taking part will be contacted approximately 4 months and 12 months after your initial home visit / recruitment group session by the research team to complete questionnaires, either by post or on the internet | To maintain engagement, encourage retention and thank foster carers, trial participants will receive a £10 high street voucher on completion of the 3 month and 12 month questionnaires. Contact details will be collected during recruitment and will be verified by provider agencies as the trial continues. **Participants will be reminded via text and email when a questionnaire will be posted to them and will also receive a newsletter updating them on study progress at 9 months post-recruitment. Participants who do not respond to postal follow-up will be contacted with the offer to complete their follow-up questionnaires via telephone if**  **they wish.** |
| **You will receive a gift voucher**  **at the follow-up consultations to say thank you for taking part, and any travel expenses will be reimbursed** | **Follow-up assessments will be carried out either by telephone or in person at approximately 2 and 6 months**. The schedule of enrolment, interventions and assessments is shown in line with the SPIRIT 2013 recommendations in Fig. 2 [24]. Participants completing follow-up assessments will be given incentive vouchers to encourage them to engage with follow up. |
| You will not be paid any expenses for your involvement in this study**, however a five pound gift voucher will be sent to you with your 18 month questionnaire** - these can be completed and returned by post or completed on your computer or smart phone (you can pick your preferred option) | Plans to promote participant retention and complete follow-up {18b}  Active measures to minimise loss to follow-up of women  include:  1. Recording at the outset women’s email addresses and mobile phone numbers, their preferred method of contact (for follow-up contact) and their preferred method of completion of questionnaires.  Questionnaires can be completed online (via an  email link) or in paper format and returned by post.  2. **Participants who do not return their questionnaires within 3 weeks will be sent up to three reminders using a variety of methods (post/email/ text message dependent on participants preferred method). The third reminder will be by telephone where the researchers will aim to gather, at a minimum, the primary outcome data during the call**.  3. Response rates to the self-reported questionnaires will be monitored to ensure they remain above 80% (the level assumed in the sample size calculation). If response rates are seen to drop, the team will discuss appropriate actions with the project management group. Relevant action may include phone calls at different times of day or asking women to only complete the primary outcome measure. |
| You will be asked to send the questionnaires back to the study office, in the prepaid envelope provided. - When we receive  your completed 15 month questionnaire,  we will send you a token of appreciation for your help. If you don’t want to receive this gift, please let us know on the consent form. - You may be sent this questionnaire by post (or email if you prefer) to complete at in the convenience of your home - | Plans to promote participant retention and complete follow-up {18b}  **We offer and use all methods of delivery and collection of questionnaires and reminders including use of research teams for time points associated with hospitalisation, post, e-mail, web-based and SMS text, taking into account each participant’s stated preferred means of receiving and completing the measures** (recorded on the participant contact preference form). **We will send up to three reminders to participants by post, email, phone or text message, taking into account any preferences they may have for mode of communication.**  **We will send a small token of appreciation (gift**  **Voucher (s) of modest value up to £15**) to participants on receiving their completed follow-up questionnaires, unless they opt out on the study consent form. |
| Everyone in the study will also be sent questionnaires by post at approximately 3, 6, 12, 18, 24, 30 and 36 months **after you join the study up to two reminders may also be sent if we don’t hear back from you....A self-addressed envelope will be provided for your convenience** – As part of this study, we will perform assessments at the same time of these check-ups | Data will be collected from the following routine visits; 3, 6, 9, 12, 18, 24 and  36 months post initial TURBT (or second TURBT if required). |
| We will provide a FREEPOST envelope for return of the annual questionnaires to the *[trial name]* Study Office, a reminder letter may also be sent if we don’t hear back from you. It is estimated that each questionnaire will take approximately 15 minutes to complete but you can take as long as you need. We realise completing the questionnaire will take a little time and to recognise this we will provide all participants a £25 gift voucher at the start of the trial and another £25 voucher at the end to thank you your support - We in tend to maintain interest in the trial by publication of  newsletters at intervals for staff and collaborators. - At the first visit your dentist will examine your teeth and gums to record information about your dental health. Every effort will be made to include this as part of your routine dental check up | All participants will be given a £25 gift voucher at the start of the trial and another £25 voucher at the end in acknowledgment of the time that participation in the trial will entail and to thank them for their support. |
| If you agree, we will send you a text message reminder 48 hours before surgery to undertake these tests. - At 4 weeks after the operation you will complete the last section of your paper diary and send the completed diary by pre-paid post to the Study Office in Aberdeen - The local research team will provide you with a baseline questionnaire which can be completed in the hospital or in the convenience of your home and returned in the pre-paid enveloped provided. - On return of your 3 month and yearly questionnaires we will send you a token of appreciation for your time spent on completing the questionnaires. Please let us know, on the consent form, if you don’t wish to receive such a token. | **Questionnaires and up to two reminders will be sent to participants by post**. **Non-responders to the 12-month post randomisation questionnaire will be contacted by phone for a short interview to capture the primary outcome** - **participants will be sent a voucher (of modest value) as a token of appreciation for completion and return of the 3-month and follow-up questionnaires** - participants will be sent the follow-up questionnaires directly from the Study Office in Aberdeen and asked to return the completed questionnaires in prepaid envelopes - We intend to maintain interest in the study by publication of *[trial name]* newsletters at intervals for staff and  collaborators |
| Every week for the 12 months of the trial we will send a reminder via the app, asking if you have had a fever or respiratory symptoms since the last time you responded - The blood collections at 6 months and 9 months may be done by self- administered finger prick blood spots with kits provided to you by the study team. This means you could collect the sample at home yourself instead of at a site study visit. If this is your preferred way of providing your sample we will also ask you for your home address so that we can mail the in-home collection kits to you - | Participants will be asked weekly if they have been unwell since the last contact using a smartphone application designed for the trial (Trial Symptom Tracker, WeGuide) **and/or by contacting the participant by telephone, text**  **message or email** |
| Two questionnaires will be sent through the post. They will ask you your views about your health and quality of life. **Please send them back in a pre-paid envelop** - **They can be done in one day or divided over a few short visits If you have left hospital we can travel to see you in a convenient place for you.** The test will be - arranged on a day(s) which suits you - We will reimburse mileage or public transport costs for any research visits | Trial participants are not paid to participate in the trial but are paid travel expenses for the follow-up visits. - As there is a low number of participants to be recruited, the trials unit are  able to liaise regularly with each site following randomisation to ensure all follow-up data are collected |
| Travel expenses will be provided for attending additional visits to increase the dose of telmisartan - We will try to arrange the study visits at the same time as your clinic visits | Follow-up visits will be designed to fit with routine hospital visits where possible. The study  will also allow a 2 week window on either side of the scheduled follow-up visit date to ensure flexibility. **Individual patients will be sent reminders about follow-up visits by the research nurses provided they have agreed to receive them**. If any of the trial patients  are lost to follow-up, contact will be attempted through the research nurse and lead investigator at each centre |
| We will ask you to give us multiple forms of contact (e.g. email, mobile / landline, and postal address) so that we can contact you  for follow-up assessments and check you are the same person - Only for the intervention group - We will send reminders to visit the website over the following 24 weeks (you can opt out of this if you wish | **Twelve and 24 weeks after randomisation, all participants are sent an email reminder to complete the follow-up measures**. At 24 weeks, the email content will vary depending on whether participants have been randomised to either £10 or £20 reward and to the reward being conditional (ie, dependent on completion of follow-up questionnaires) or unconditional (ie, offered with the initial request for follow-up data).  To maximise follow-up at the primary outcome point  we will:  1. only randomise participants once baseline assessment measures are completed  2. include detailed explanations in our recruitment materials to explain to participants why data completion at follow-up is so important  3. require email, telephone and postal contact details at registration so we have multiple methods of contact for follow-up  **4. send participants up to three automated email reminders at 5-day intervals, followed by text, telephone and/or postal requests**  **5. incentivise completion of follow-up measures by paying participants shopping voucher(s) at each time point.**  To increase overall acceptability and participation rates, we will inform RD participants that they will be able to access toolkit modules after the final follow-up. |
| You will do it during a face-to-face appointment with our researcher. This appointment will last about one hour and happen at a time and place that is convenient for you - We do not expect you will have any expenses from taking part in our study. **If needed, we can pay for a taxi to take you and your family member or friend to and from the course.** We will also provide refreshments for you. All participants will receive a £10 shopping voucher for each of the questionnaires they do by post or in person. This is to thank them for their time and effort. Therefore, each person who takes part in our study can get up to £30 | A number of evidence-based strategies were used to maximise retention of participants in the trial. This included patient and SO participants receiving a £10 shopping voucher after each follow-up assessment that they completed and delayed access to SAFE for the TAU arm. **Unless a participant formally withdrew consent to participate in the trial, HES data on them were requested and up to three attempts were made to contact patients or SO participants each time a follow-up assessment was due. The research worker also contacted participants by telephone approximately 2 weeks after the 6-month questionnaires were posted.**  **When a patient participant did not complete a follow-up assessment, an attempt to monitor SAEs was made by sending their GP a letter asking them to inform us if the patient was no longer alive and the**  **circumstances of death** |
| We will offer you £5 as compensation for your time at each follow up: for the 1 month questionnaire and the 12 month questionnaire. You will be compensated for your time for returning your 12 month sample. | At 4 weeks and at 1year, postal questionnaires will be sent to all participants to collect self-reported data. **Non responders will be contacted by any method the participant agrees to at enrolment (post, email, text message, telephone call).** Participants can directly enter self-reported outcome data via a web-based data entry form. Paper based self-reported outcome data or self-reported data collected by telephone will be directly entered into the web-based data entry form by a trial assistant blinded to treatment allocation.  Participants will be sent a £5 unconditional incentive with each postal request, that is, when sending the 4 weeks questionnaire and 1year test and questionnaire. **Participants who return the test sample will be sent £20.** |
| So we know how you are getting on we will also send you a questionnaire, this questionnaire will take approximately 30 minutes to complete. There will be an option to complete this questionnaire online. | - each woman will be sent a postal or online questionnaire (as preferred by each woman) for col lection of data on secondary outcomes. **Text reminders for completion will be sent as appropriate, with the option for telephone completion in the event of a delayed response to ensure a high response rate.** |
| You will not be compensated for your involvement in this study but **reasonable travel expenses to the hospital for any appointments that are outside of routine care can be reimbursed.** The local research team will provide further details about this - Whichever treatment you receive, you will be asked to complete an email or paper follow up questionnaire at six months; twelve months and eighteen months after warhysiotherapy or surgery - All patients participating in this study will attend follow up visits and be monitored at their local hospital as they would normally | Follow up for study purposes will be by patient self-reported questionnaire completed using a web-based data collection system. The option of being able to fill out the follow-up questionnaires in a hard copy and returning via post will also be available. Non-response will be minimised through use of multiple reminders  such as web-based messages, phone calls and texts |
| We ask that you complete and return this to the Research team in the stamped addressed envelope provided. We will remind you with a letter and via telephone if you do not return your questionnaires. - **Travel expenses for your research appointments will be refunded by the**  **Research team. We are not able to pay travel expenses for your physiotherapy treatment** - | All participants are invited to attend a face-to face clinic appointment at 6 and 12 months. This is arranged by the researcher at each site. However, if a participant is unable to attend the clinic appointment, they are mailed a questionnaire that contains the primary outcome, all self-reported items and a participant completed version of the Client Service Receipt Inventory, but excludes the  physical assessment. If the questionnaire is not returned within 2weeks, then a second copy of the questionnaire is sent by the *[trial name]* Trial Office as a reminder. If this is not returned within a further 2weeks, then the *[trial name]* Trial Office carries out a reminder phone call. **After another 2 weeks, if the questionnaire has not been returned, then the *[trial name]* Trial Office will attempt to contact the participant by telephone and collect core outcomes consisting of the primary outcome (ODI), pain troublesomeness rating, whether they are on a waiting list for spinal surgery, EQ-5D-5L, self-rated walking ability, falls and falls-related fractures, self-reported exercise adherence, and a brief version of the Client Service Receipt Inventor** |
| Reasonable travel expenses to study-specific appointments can be reimbursed. - you can choose to complete these online or by post. The research team in your hospital will have access to your answers. We may get in contact by post, email or phone to remind you to  complete the questionnaires | These will be posted or emailed by the Clinical Trials Unit and can be completed by post or online. **If participants do not respond within 2 weeks of sending the questionnaire, a reminder will be sent. Following this, if still no response, the central trial team may telephone the participant to collect the required data.**  - Reasonable travel expenses for any visits additional to normal care will be reimbursed on production of receipts, or a mileage allowance provided as appropriate. **Participants attending focus groups will receive a £20 shopping voucher to thank them for their time and participation, in addition to travel reimbursement as above**. |
| If for any reason you miss your clinic appointments or do not respond to our postal questionnaires we will contact you by phone on a maximum of 2 occasions. We can then post the questionnaires for you to complete and return in the stamp addressed envelope provided. You will not have to make any extra visits to your doctor or the hospital over and above those needed for your normal care - -**All postage will be pre-paid** - you will not be asked to attend any appointments which you would not have to for your regular clinical appointments as we will aim to schedule these alongside your clinical appointments. | Data will be collected alongside routine clinical appointments at each site - Data will be collected from participants at 6 and 12months  (if applicable) from date of surgery with a target of ±1month, at their routine NHS check-up appointments. If participants do not attend their follow-up appointment, they will be contacted by telephone, and, if appropriate, sent the questionnaires to complete. The study team will attempt to telephone these participants on up to two occasions. If these methods fail, we will categorise the participant as a ‘nonresponder’ for that time-point only - Follow-up data collection via telephone, and postal questionnaire data collection options have been added to minimise the need for participant hospital attendance. The study team will attempt to contact these participants on up to two occasions to remind them to complete the questionnaires. If these methods fail, we will categorise the participant as a ‘non-responder’ for that time point only. Qualitative data will now be collected using telephone interviews for all group |
| We can reimburse some travel costs if these are additional to your routine medical care travel. - and you will be asked to complete them on-line (a paper version is also available).You will be sent e-mails and text reminders to complete these, or reminders in the post if you have chosen to complete paper versions. -A member of the *[trial name]* team may contact you by telephone (up to a maximum of 5 times over the 6 months that you are in the trial) if you have not completed these questionnaires and to see if you require any assistance. - **We would normally expect all trial visits to coincide with routine standard care. If you do incur any additional parking expenses for travel, please talk to the research team about completing a claim form** | Loss to follow-up: If a participant moves from the area, every effort should be made to follow the participant up at another participating trial site and for this new site to take over the responsibility for the participant. If a participant is lost to follow-up at a site, every effort should be made to contact the participant’s GP to obtain information on their current status. - Those participants who state at recruitment that they cannot or do not wish to complete these questionnaires online will be sent the  paper version by the *[trial name]* trial office and requested to complete the questionnaires and to return them in a prepaid envelope. - participants will be requested to complete PROM questionnaires and resource-use diaries electronically using LimeSurvey every week and every 3 months, respectively. A paper version will also be available for participants to complete, which can be returned using prepaid  envelopes. Text messaging and email reminders to participants (who have consented to provide their telephone numbers and email addresses) who have not returned the completed PROM questionnaires and resource-use diaries will be used to maximise the completeness of the data.  The *[trial name]* trial office may also call participants if there are a number of outstanding PROM questionnaires and  resource-use entries not completed by the participant |
| **Do not communicate the same information regarding plans use a strategy to promote participant retention** | |
| **PIL content** | **Protocol content** |
| Patients will be reimbursed for any additional travel costs incurred from trial related visits  to the hospital (outside of standard medical care). To claim, please contact your local researcher - additionally those in the control group have choice of phone data collection for follow up if they do not need to attend the clinic for another reason | The questionnaires can be filled in online. For this a personal email with the invitation and access code will be sent to the patients by mail. Patients not willing or unable to complete the online questionnaires will receive identical paper questionnaires at their home address, accompanied by a free return envelope. |
| Most of the treatment you will receive will be the same whether you were in the study or not but you will also be asked to complete a questionnaire about your quality of life before you have your treatment, and 1, 6, 12, 24, and 36 months after wards | Appropriate strategies to promote participant retention will be considered and implemented by the Trial Management Group (TMG) as required. |
| Filling out the diary will take about five minutes at the end of each day. This can either be on paper form or over the internet.  We will send you a brief text message or phone you every week to remind you complete this. - Reasonable travel expenses will be reimbursed for all research visits | Every attempt will be used to collect full follow-up data on all women. In particular, participants will continue to be followed up even after protocol treatment violation. It is thus anticipated that missing data will be minimal |
| You will be asked to complete these, and then post them back to BCTU in the freepost envelope they will send you | participants will be sent a newsletter thanking them for their support and informing them of the trial results |
| Although you will not be paid to take part in the study, we will cover your expenses to attend study visits | All patients in the RCT and cohort study will be contacted every 6 months by phone to enhance follow-up rates and to collect data about any new relevant developments and ensure continuation of consent and patient safety. |
| We are unable to pay you for participating but we will reimburse your travel expenses to and from the hospital for all visits | In the event of participants being unable to attend for scheduled visits because of other intercurrent issues, they are asked to attend at the closest possible date, whenever possible within 2 weeks of the due date. Where a participant cannot attend their week 40 study visit and investigations, they are asked either to undertake this visit up to 3 weeks before the due date or, alternatively, to continue the study medication until the scheduled investigations can be undertaken (whenever possible within 4 weeks of the planned date) |
| The Sponsors of this study (Imperial College London) will pay your hospital to cover the costs of your participation in this study. Yes - You are able to claim the travel costs (e.g. bus / train / tube fare or parking costs and petrol) of your hospital visit for your scan at 2 to 3 weeks following your surgery | Telephone or online review (according to patient preference) will be performed 1 week after surgery or at discharge, and at 90 days - Routinely scanning patients only once reduces cost, and limits the time and inconvenience to the enrolled study subjects (ethical consideration, improve recruitment, and reducing drop-outs and loss to follow-up) |
| Whenever you visit the hospital for a visit that would not be part of your normal routine care (This will happen during your maintenance treatment) you will be able to claim £5 towards your travel expenses - Most of your visits and assessments will be as you would have in routine care if you weren’t taking part in a trial (However some visits and assessments are additional) | All efforts should be made to contact the patient’s GP to assess their condition, if a patient fails to attend a clinic or cannot be followed up at site. |
| We will offer you a total of £20 in vouchers as a thank you for taking part. We will offer £10 for completing the first set of questionnaires  and another £10 when you complete the third  set of questionnaires at 12 months  - Initial postal questionnaire sent with a pre-paid return envelope | all participants will be provided with study progress updates at 3 and 9 months via a newsletter to maintain engagement with the trial and encourage response rates of follow-up questionnaires. The newsletter does not provide any detail on the *[trial name]* intervention - All participants complete self-reported outcome measures in the form of questionnaires (IPSS, ICIQ-UI-SF,EQ-5D-5 L and B-IPQ) at baseline (postal), 6 and 12 months (postal, online or phone) post enrolment. Participants are sent one reminder to return their baseline materials, and up to three reminders to return their 6- and 12-month questionnaires. |
| The sponsors of this study will pay [enter name of hospital department]to cover the costs of your participation in this study - - You will have one additional hospital visit at 6 weeks for assessment of your ulcer. Other than the 6-week leg ulcer clinic visit, the number of appointments is the same as treatment for your ulcer outside the study | Similarly, efforts will be made to obtain complete follow-up for all randomised participants (irrespective of whether or not they underwent allocated treatment). For those participants unable or unwilling to attend follow-up appointments, home-visits or follow-up by community nurses may be considered |
| We would be very grateful if you would complete them and post them back in the provided prepaid envelope.  *[not communicated in the other corresponding PIL]* | Patients will be followed up as per routine clinical practice and typically at 3, 6 and 12 months and annually thereafter. Patients may be seen at other times as clinically indicated. |
| Study visits will take place every three months and should normally be scheduled to take place at the same time as your normal hospital visit | Scheduled study visits are designed to fit with routine hospital visits where possible. - If any of the trial participants are lost to follow up contact will initially be attempted through the PI or delegated research staff at each centre. If the lead investigator at the trial centre is not the participants usual clinician responsible for their specialist care then follow up will also be  attempted through this latter clinician. Where these attempts are unsuccessful, the participants GP will be asked to contact the patient or the participants’ family to provide follow up information to the recruiting centre. This information will be included on the Patient Information Sheet. Wherever possible, information on the reason for loss to follow up will be recorded. |
| Completing 6 short questionnaires on your muscle symptoms every 2 months. There are a few ways in which you can choose to complete the questionnaires; via the web, verbally over the phone, mobile phone app or conventional paper form - | In the seventh week of each 2-month treatment period, participants will receive reminders to alert them that follow-up data collection is approaching. Symptom scores  on the VAS will be collected daily in the eighth week of each treatment period. Participants can choose to receive daily reminders on each day their data is due to be collected. Non-responders will automatically receive a  reminder from the trial team after 24hours of the due date |
| The study can pay you back for all reasonable costs for travelling to your study appointments (e.g. car parking and petrol or other transport costs). Please make sure to ask at the clinic | For participants who become unwilling or unable to attend study clinic visits,  local research staff telephone the participant (or interview their local doctor or relative). All efforts are made to continue to follow up such participants, with those being followed  remotely encouraged to continue to provide central blood samples |
| we will reimburse reasonable expenses for travelling to your *[trial name]* appointments. Please make sure you ask about this at the clinic. Taxi travel can also be provided if needed | Patients will be asked, if willing, to provide details of a friend or relative living at a different address who may be contacted in the event of loss of contact. They will also be asked their preference for receiving questionnaires (by email or telephone) and email addresses will be recorded. - Trial-specific questionnaires will be administered either electronically (for completion on participants’ own computer) or by telephone (according to the preference of the participant) while EQ-5D and VFQ-25 questionnaires will be administered electronically, by phone or on paper at set intervals as detailed in section 4.7. The *[trial name]* trial website will provide information regarding the progress of the trial and newsletters will be sent to participants updating them about the progress of the trial - Failure by participants to complete electronic questionnaires will result in emailed reminders being sent, followed by LCC research nurses undertaking telephone follow-up if there is still no response. |
| We are not able to pay travel expenses for you to attend your follow-up sessions, however any research questions asked will be as part of your routine out-patient follow-up appointments, via email, or over the telephone | Data will be collected from participants and proxies via questionnaires and CRFs that will be returned to the central trial office in Oxford, via post using a pre addressed freepost envelope, NHS email as appropriate or directly into an online secure database |
| if you were required to attend any additional visits, travel costs for these would be reimbursed. All postage and telephone costs will be pre-paid | Questionnaires will be sent to participants by post from the trial office and returned using a pre-addressed, prepaid envelope. If participants have not responded within 14 days of posting, the trial team will attempt to telephone the participant on up to two occasions to remind them to complete the questionnaires. If required, a second postage of the questionnaires will be provided if requested by the participant during these follow-up telephone calls. If participants wish not to complete the questionnaires, they will be provided with the opportunity to complete the UCLA Activity Scale and EQ-5D-5L questionnaires over the telephone. If these methods fail, the participant would be categorised as a non-responder for that time point only. In an effort to find evidence-based techniques to improve retention and recruitment to RCTs, a study within a trial (SWAT) will be undertaken. This will examine whether there is a difference in questionnaire response rate by printing the UCLA Activity Scale on pink rather than white coloured paper at the 6month time point. Results of this SWAT will be reported separately to the ‘host’ trial. |
| Visits would be held at your school/college unless you leave school during the study, in which case we will arrange follow up visits in the community | Meningitis Now provided funding for a prize draw to facilitate participant retention. |
| You will not be paid for taking part in this trial. However, we will reimburse you for any travel expenses you incur for visits resulting from your participation in the trial, as by participating in the trial, you will be asked to attend more clinics at your GP surgery. If independent travel is difficult for you and might preclude your participation in the trial, you can contact the trial team directly who will discuss alternative arrangements for travel with you | Where questionnaires are not validated for use on a tablet computer, or where individuals are not comfort able using one, paper copies will be made available for completion |
| Your *[trial name]* appointments will be planned at the same time as your usual appointments. If  this is not possible, we can carry out these over  the phone or pay back any additional travel  expenses | participants will be contacted at around 8 weeks postdelivery and asked  either in person/over the phone/by post/or via email to complete patient questionnaires and arrange for collection of the loaned BP monitor if not returned |
| You will receive £40 in shopping vouchers for completing the study | At 3 and 9 months post-baseline all participants are posted a questionnaire booklet and a freepost envelope to return the completed booklet to the CTU. A £20 shopping voucher is mailed to participants on CTU receipt of the completed booklets at both 3 and 9 months. To increase response rates, motivational postcards are mailed to participants before the follow-up questionnaires are sent out. Up to two reminder letters are issued (and a further three telephone calls as required) to remind participants to return the questionnaire booklets, and the option of the participant telephoning a member of the research team to aid completion of the booklets is offered. Participants are given the option to just complete the key questions about smoking behaviour if the questionnaire booklet is not returned to the CTU within 2 weeks and to submit these responses by email, phone or text if preferred to maximise follow-up data on key outcomes. - , and a freepost return envelope to be sent to the CTU. To maximise data completeness, participants scheduled to receive an accelerometer were sent a standardised letter from the CTU, 2weeks before receiving the accelerometer, advising them that they would shortly be receiving the device, and asking them to inform the CTU if they were unable to wear it. A letter was sent to participants who did not object to wearing the device, 3 days into the 10-day recording period prompting participants to start wearing the device if they had not already done so. Up to two reminder letters and a follow-up phone call were made to participants if they did not return the accelerometer. |

**2.4 Mention of Patient and Public Involvement in the “Adult PILs”**

In terms of Patient and Public Involvement (PPI), 7.6% (n=7/92) of the PILs communicated that there was PPI involvement in the trial.

| **Table 5: Mention of PPI involvement in the trial in the PIL** | |
| --- | --- |
|  | **Number of PILs (% out of 92 PILs)** |
| **Yes** | **7 (7.6%)**  **Examples from PILs**  “A group of patients and members of the public helped to develop this research topic and the research questions that should be asked. The group helped to design the study and develop this leaflet. They will continue to be involved throughout the study”.  “A Patient and Public Involvement (PPI) group have contributed to the design of the trial and will continue to be involved throughout the study, overseeing the study progress”.  “Our study team includes *[disease specific charity]*, a charity that supports patients and families”.  “People with *[disease]* and their carers are part of the study team”  **Note:** These 7 PILs correspond to 7 protocols of which 5 report PPI involvement (71.4%) |
| No | 85 (92.4%) |

**Abbreviations**

Clinical Trial Units (CTUs)

Online Resources for Research in Clinical triAls (ORRCA)

Participant Information Leaflets (PILs)

Standard Protocol Items: Recommendations for Interventional Trials (SPIRIT)

Patient and Public Involvement (PPI)

**References**

1. Gillies K, Kearney A, Keenan C, Treweek S, Hudson J, Brueton VC, et al. Strategies to improve retention in randomised trials. Cochrane Database of Systematic Reviews. 2021(3).

2. Kearney A, Daykin A, Shaw ARG, Lane AJ, Blazeby JM, Clarke M, et al. Identifying research priorities for effective retention strategies in clinical trials. Trials. 2017;18(1):406.

3. Online Resources for Research in Clinical trials. Retention Research Domains. [internet] [Accessed 13/01/2022] [Available from: <https://www.orrca.org.uk/Uploads/ORRCA_Retention_Domains.pdf>.

4. Chan A-W, Tetzlaff JM, Altman DG, Laupacis A, Gøtzsche PC, Krleža-Jerić K, et al. SPIRIT 2013 statement: defining standard protocol items for clinical trials. Annals of internal medicine. 2013;158(3):200-7.

5. Chan A-W, Tetzlaff JM, Gøtzsche PC, Altman DG, Mann H, Berlin JA, et al. SPIRIT 2013 explanation and elaboration: guidance for protocols of clinical trials. Bmj. 2013;346.
